# Screening for foot problems in children: is this practice justifiable?

**DOI:** 10.1186/1757-1146-5-18

**Published:** 2012-07-24

**Authors:** Angela Margaret Evans

**Affiliations:** 1 AUT University, North shore campus, Akoranga Drive, Auckland, 29006, New Zealand; 2 University of South Australia, City east campus, North Terrace, Adelaide, 5000, Australia

**Keywords:** Screening, Paediatric, Foot, Flatfeet, Children

## Abstract

Podiatry screening of children is a common practice, which occurs largely without adequate data to support the need for such activity. Such programs may be either formalised, or more ad hoc in nature, depending upon the use of guidelines or existing models. Although often not used, the well-established criteria for assessing the merits of screening programs can greatly increase the understanding as to whether such practices are actually worthwhile. This review examines the purpose of community health screening in the Australian context, as occurs for tuberculosis, breast, cervical and prostate cancers, and then examines podiatry screening practices for children with reference to the criteria of the World Health Organisation (WHO). Topically, the issue of paediatric foot posture forms the focus of this review, as it presents with great frequency to a range of clinicians. Comparison is made with developmental dysplasia of the hip, in which instance the WHO criteria are well met. Considering that the burden of the condition being screened for must be demonstrable, and that early identification must be found to be beneficial, in order to justify a screening program, there is no sound support for either continuing or establishing podiatry screenings for children.

## Background

Children’s foot conditions are a frequent cause of parental concern, and as a result, regularly present to a range of medical and health care clinicians. Whilst a variety of issues may arise, (e.g. warts, curly toes, footwear, ingrown nails), foot posture and especially flat feet is a dominant concern. Indeed, the foot has been found to be the most common musculoskeletal region presenting to general practice in the UK [1]. However, the level of presenting concern of foot posture problems in children may not necessarily reflect a demonstrated level of frank pathology. Given that paediatric flatfoot has been cited as the most common presentation in paediatric orthopaedic clinics [2,3], and that the consensus from epidemiological findings is that paediatric flatfoot reduces with age and is mostly asymptomatic [4], the rationale for screening programs becomes curious.

This discussion paper has been generated in response to the common practice of podiatrists attending pre-schools and schools to offer podiatry screenings for children. Concerns relating to this practice have been reported both formally and informally from members of the public, and medical and health professionals. In response to such concerns the Australian Podiatry Association (South Australia), developed guidelines for screening in 1998, with revision in 2008. A summary of these guidelines is provided in Table 1.

**Table 1:** A summary of the guidelines from The Australian Podiatry Association (South Australia) in 2008 for podiatrists’ visits to children’s centres/preschools for paediatric foot screening

**Section**	**Area covered**	**Basic content**
1	General	Purpose of the document and definitions
2	Policy	Australian Podiatry Association (South Australia) policies for members
3	Guidelines	Obtaining consent from all relevant bodies
4	Protocol	Outline of the visit format and content
5	Appendices	- consent form for parents
		- report form of examination findings for parents

Criteria for screening programs, regardless of the condition/s to be detected are well recognized [5], although often overlooked or not considered. This paper aims to critically review the issue of foot screening for children, and to establish whether such programs are indicated, based upon previously developed criteria for screening programs (WHO) which have been used for the ocular condition, amblyopia [6].

### Lessons from previously reported foot screenings

In 1967, Shapiro and Rhee conducted screenings at a time when unsubstantiated opinions about foot conditions (e.g. “many of the foot disorders in adults begin in childhood”) were accepted, rather than tested [7]. As a result, the findings must be seen in that light. This series of screenings examined 8,995 children over a two year period, and found the most frequently encountered problems to be postural or orthopaedic, and most frequently this was flatfeet. Given that more than half of the subjects were less than six years old, this finding is now seen as an expected part of development, but at the time was seen as justification to continue screening (and subsequently examining ‘positive’ cases further, and instigating treatment).

Almost 40 years later, El et al. conducted a screening in 579 school children (mean age 9.23 years) and found significant and inverse correlation between flatfeet, hypermobility and age. These authors concluded that flexible flatfoot and joint hypermobility both reduce developmentally [3]. In the same year Pfeiffer examined 835 children aged three to six years and found increased flatfoot posture in boys, in younger children, and in overweight or obese children. Pathological flatfoot was detected in less than one percent of subjects, yet 10% were being treated for their flatfoot posture, giving rise to the conclusion that 90% of treatment was unnecessary [8].

## The principles of screening

The World Health Organization (WHO) defines screening as: “the presumptive identification of unrecognised disease or defects by means of tests, examinations or other procedures that can be applied rapidly” [5].

Further to this, the WHO stipulates that screening is intended for all people, in an identified target population, who do not have symptoms of the particular disease or condition. The screening process can then potentially identify: a pre-disease abnormality, early disease, or disease risk markers.

The aim of screening for a disease, or a risk marker for a disease, is to reduce the costs of the disease in the community–including incidence of disease, morbidity or mortality. This is achieved by intervening to reduce individual risk of the disease, or detecting the disease earlier than is usually the case in the absence of screening, and hence improving disease outcome.

Clearly the impact of the disease or condition must be demonstrable, and early identification found to be beneficial, in order to justify any screening program. There must be evidence that early diagnosis and treatment increases the chances of successfully treating or managing the disease.

Whilst screening can reduce the risk of developing or dying from a disease, it does not guarantee that disease will not occur, or if it occurs, that it can be cured. A ‘positive’ screening test identifies people who are at increased likelihood of having the condition and who require further investigation to determine whether or not they have the disease or condition.

As screening has benefits, costs, and also potential harms, there is an ethical obligation to maximize benefits and minimise harm; and the overall benefits should outweigh any harms that result from screening. In addition, when community resources are used to fund screening there should be community consensus that the benefits of screening justify the financial expense of undertaking the screening.

## Health screening in Australia

Undetected but untreated, tuberculosis, and cancers of the breast, cervix and prostate gland result in death and suffering. Early treatment gives the best outcomes for survival, and screening has been demonstrably effective in reducing incidence, morbidity and death [9].

As outlined within the Australian Health Ministerial Advisory Council (AHMAC) Australian Population Health Development Principal Committee Population Based Screening Framework (2008), in Australia, screening programs currently occur for: tuberculosis; breast, cervical, and bowel cancers [10]. It is pertinent here, to briefly review each condition.

### Tuberculosis (TB) screening: reduces incidence, illness and deaths

Most individuals infected with *Mycobacterium tuberculosis* remain asymptomatic, but there is a 10% lifetime risk of developing clinical illness, sometimes many years after the original infection. The World Health Organization (WHO) declared tuberculosis a global emergency in 1993, and recent reports have reaffirmed the threat to human health. About 1,000 cases of TB are notified to Australian health authorities annually. The yearly notification rate for TB has stabilised at approximately 5 to 6:100,000 since 1985.

**Figure 1:**
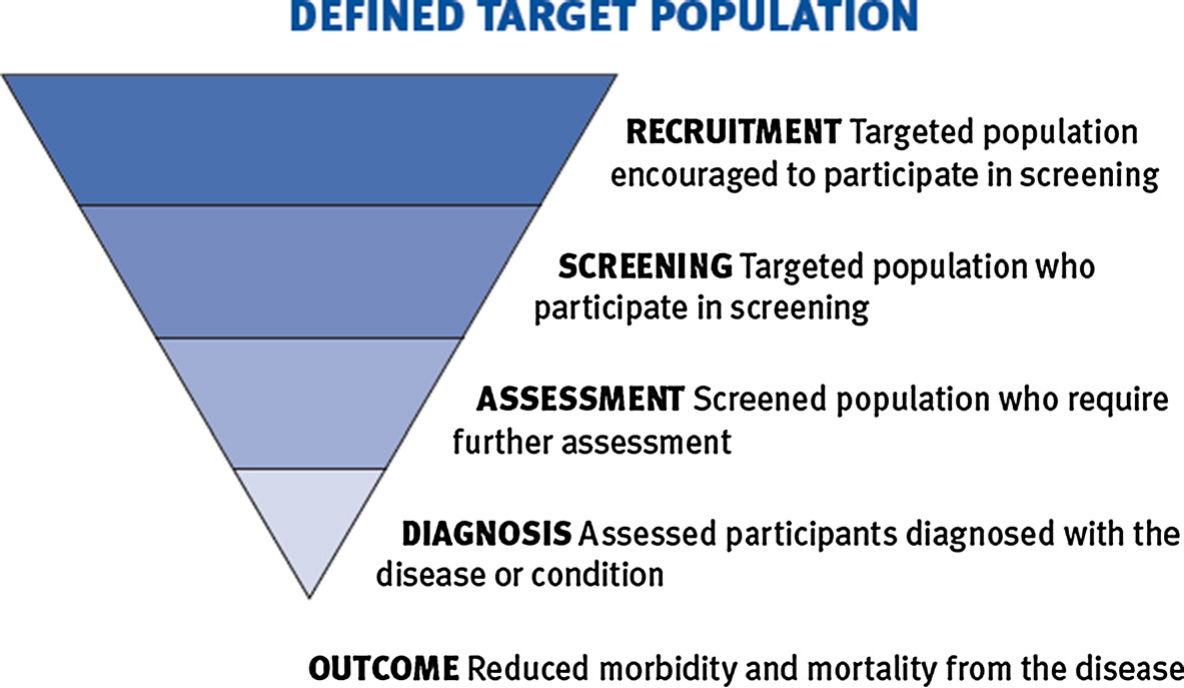
**The framework used for population based screening programs in Australia.** Reproduced from: Population Based Screening Framework, Australian Health Ministerial Advisory Council (AHMAC), 2008.

In Australia, most TB cases (> 80%) occur in people born overseas, particularly in Asia, eastern Europe, the Pacific Islands, and sub-Saharan Africa. The rates of TB in the overseas-born population have been slowly increasing over the past decade. Rates of TB are also high in Aboriginal and Torres Strait Islander people and in Papua New Guineans living in some parts of Australia. Patients with impaired immunity are at high risk of developing active TB if they are infected with *M. tuberculosis*. Screening programs in Australia now target those at high risk, which includes those in contact with affected patients (Figure 1).

Vaccination programs for TB uses the BCG (Bacille Calmette-Guérin) vaccine, and whilst globally, many BCG vaccines are available, all are derived from the Institut Pasteur, which was first used in humans in 1921. BCG is primarily intended for children as meta-analyses have found the protective efficacy for preventing serious forms of TB in this group is over 80%. Protective efficacy in all age groups is about 50%.

The Australian cancer council has summarized the targeted approach to screening for breast, cervical and bowel cancers [11].

### Breast cancer screening: reduces morbidity and mortality

Breast cancer is the second most common cancer affecting Australian women (after non-melanoma skin cancer), and the second most common cause of death in Australian women. Breast cancer screening in Australia targets women aged between 50 to 69 years, who are most affected. Early detection of breast cancer has been found to give the best chance of reducing both morbidity and mortality [11].

### Cervical cancer screening: reduces incidence and deaths

More than 730 new cases of cervical cancer occur in Australian women each year, and results in approximately 210 deaths in women annually. Since 1991 when cervical cancer screening commenced in Australia, the incidence of cervical cancer in women aged 20 to 69 years has almost halved, saving approximately 1,200 women from developing cervical cancer each year [11].

### Bowel cancer screening: reduces incidence and deaths

Bowel cancer affects 1:12 Australians by age 85 years, making it the third most common cancer (after skin and prostate cancers), and the second most common cause of cancer deaths in Australia (after lung cancer). There is a demonstrated 90% cure rate with early detection and treatment. The current screening program targets those turning 50, 55 and 65 years between January 2011 and December 2014 [11].

In 1968, Wilson and Jungner developed the WHO principles of screening [5]. These are outlined in Table 2, and expanded upon below within the context of paediatric podiatry foot (posture) screenings.

**Table 2:** The WHO criteria help to distinguish between population-based screening and opportunistic case-finding

**WHO Principles of Early Disease Detection**	
Condition	
· The condition should be an important health problem.	
· There should be a recognisable latent or early symptomatic stage.	
· The natural history of the condition, including development from latent to declared disease should be adequately understood.	
Test	
· There should be a suitable test or examination.	
· The test should be acceptable to the population.	
Treatment	
· There should be an accepted treatment for patients with recognised disease	
Screening Program	
· There should be an agreed policy on whom to treat as patients.	
· Facilities for diagnosis and treatment should be available.	
· The cost of case-findings (including diagnosis and treatment of patients diagnosed) should be economically balanced in relation to possible expenditure on medical care as a whole.	
· Case-findings should be a continuing process and not a ‘once and for all’ project.	

Reproduced from: Principles and practice of screening for disease, WHO, 1968.

## Application of screening principles to the paediatric foot

This discussion of whether the paediatric foot is a suitable target for community screening has been generated by the increasing propensity for screening programs, without adequate data to develop a clear understanding of how effective these programs are likely to be. The criteria by which a potential screening program should be judged, whatever the condition to be detected, are well recognised. These criteria are often overlooked altogether, or only considered in part, in the establishment of screening programs. Podiatry screening programs of varying nature exist in many communities, and for children, most address the paediatric flat foot. Ill-defined goals can include ‘prevention’, or the early detection and treatment of ‘foot problems’—the notion of which is inherently unsubstantiated for paediatric flatfoot [12]. Here then, we review critically whether such programs are either helpful or appropriate, by considering how well paediatric foot problems (viz. flatfoot) fulfils each of the following WHO criteria for a screening program.

### Condition

(i)  *The condition should be an important health problem.* Prevalence studies of paediatric flatfoot have returned a wide range of estimates from 0.6–77.9%, due to differing age groups, assessment measures and population samples [4]. The findings of developmental flatfoot across age groups approximate 45% in pre-school children and 15% in children with mean age of 10 years. The rate of paediatric flatfoot may be higher with joint hypermobility [13], increased weight or obesity [14], in males [8], with specific neuromuscular [15] and genetic conditions (e.g. Down’s syndrome) [16], or with a positive family history [8]. However, reported prevalence varies among studies according to the populations examined, and the tests and criteria used. Despite being so common, there is little known about the long-term consequences of paediatric flatfoot–vital knowledge in determining the importance of the condition [17].  Whilst the premise of preventing foot problems from either existing or developing in childhood are notionally admirable, there is a lack of evidence from prospective data to demonstrate which pediatric flat feet require, and may benefit from, treatment [4]. It is sensibly accepted that painful paediatric flatfeet warrant intervention [18], but it is also pertinent to note that some paediatric foot pain may be transient and attributed to normal osseous development alone (e.g. Sever’s disease), and not attributable to foot posture alone. It is also the case that most paediatric flatfeet, especially in the first decade of life, do not present with overt symptoms. In such instances it is largely the appearance of the foot morphology which concerns parents and sometimes clinicians [19-21]. There is understandably, an impetus to avoid future problems and feared disability due to extrapolated foot pain (more commonly seen in adults), but in healthy children where flatfoot developmentally reduces with age, this is an unfounded concern. It is very important that the paediatric flatfoot is well assessed, as this provides the opportunity to filter the normal physiologic flatfeet from those related to other known associations, including joint hypermobility, overweight or obesity, or specific disease groups. The paediatric flatfoot proforma (p-FFP) provides an assessment framework which standardises both assessment and management, according to the currently available best evidence [22]. Some flatfeet, either within or continuing from childhood, will be come painful and hence require treatment. However, there is no known a priori test that definitively detects the errant cases, and hence treatment of paediatric flatfeet requires either the presentation of symptoms or clinically assessed functional deficits, to warrant intervention. In turn, any interventions need to be assessed for need, benefit and efficacy against pre-specified outcome measures.

(ii)  *There should be a recognisable latent or early symptomatic stage.* Some factors may, independent of development, predispose children to flatfeet which are not considered normal. Hypotonia, is a good example of a condition which is often detected before foot posture becomes relevant (i.e. before walking is expected) and which may be related to delayed motor milestones [23]. However, it is difficult to ascertain what proportion of infants who are detected with factors such as hypotonia, will be affected in terms of motor delays involving foot posture (stance and gait onset), as the presentation and pathway is highly variable, and generally diluted by age. Hence, even given the identification of a factor more likely (but not assuredly) to herald increased paediatric flatfoot, prediction of the need for treatment remains uncertain, making the issue of a recognizably latent period rather fraught.

(iii)  *The natural history of the condition, including development from latent to declared disease should be adequately understood* The natural history of foot posture development is well documented, and whilst assessment techniques, samples, ethnicities and age ranges have varied, there is uniform consensus that the ‘flatness’ of paediatric foot posture reduces normally with age [20,24-26]. It is not fully understood why and at what age children develop out of the physiologic flatfoot posture, although good assessment of the wider developmental status (i.e. muscle tone, connective tissue quality, strength, genetic factors, antenatal growth, obesity, gender, inheritance, shoe use) yields clinically useful indicators [27]. In order for guidelines for necessary and beneficial treatment to be effective, the natural history of normal from aberrant foot posture development needs to be known, in both healthy children and those with an underlying diagnosis (e.g. juvenile arthritis, cerebral palsy, Down’s syndrome, diabetes mellitus).

### Test

**Figure 2:**
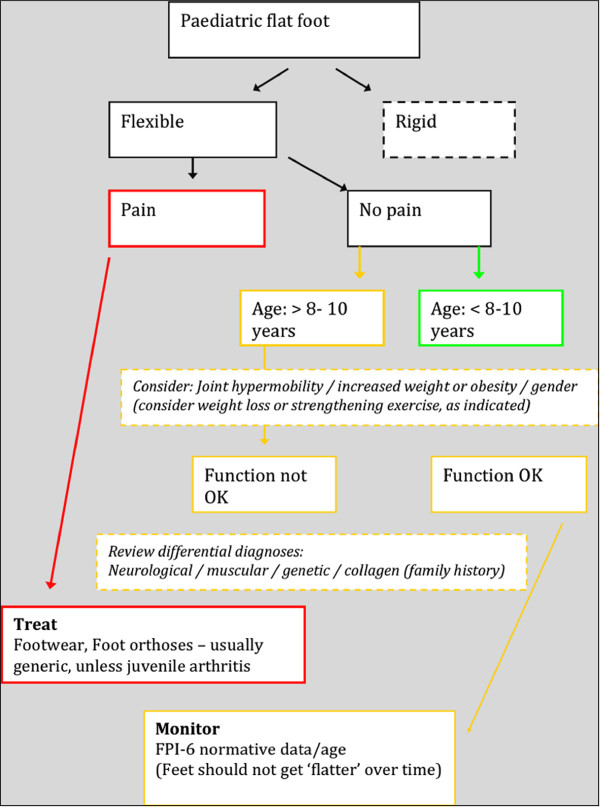
**This algorithm displays the best available evidence for assessing and managing flatfoot in children is derived from the paediatric flatfoot proforma (p-FFP).** Reproduced from: A Cochrane review of the evidence for non-surgical interventions for flexible pediatric flat feet, Evans AM, Rome K (2011) European Journal of Physical Rehabilitation Medicine 47(1): 69–89.

**Figure 3:**
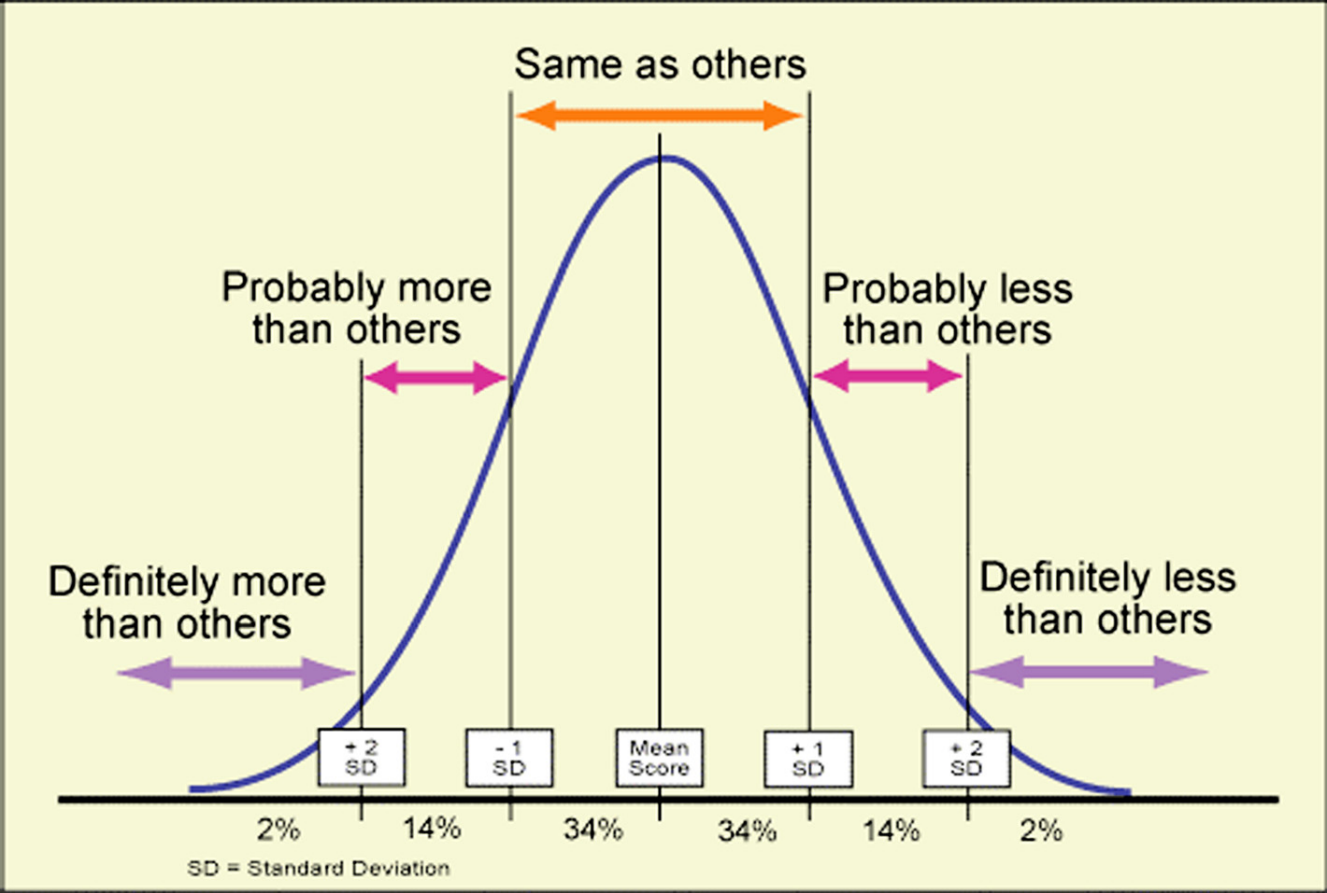
**The statistical definition of ‘normal’ is the area under the curve, which is two standard deviations either side of the population mean. This represents 95% of any normally distributed sample, such that only 2.5% are above and below these values. Reproduced from:**http://michaelsrickert.wordpress.com/2012/02/06/what-is-your-emr-bell-curve/

**Figure 4:**
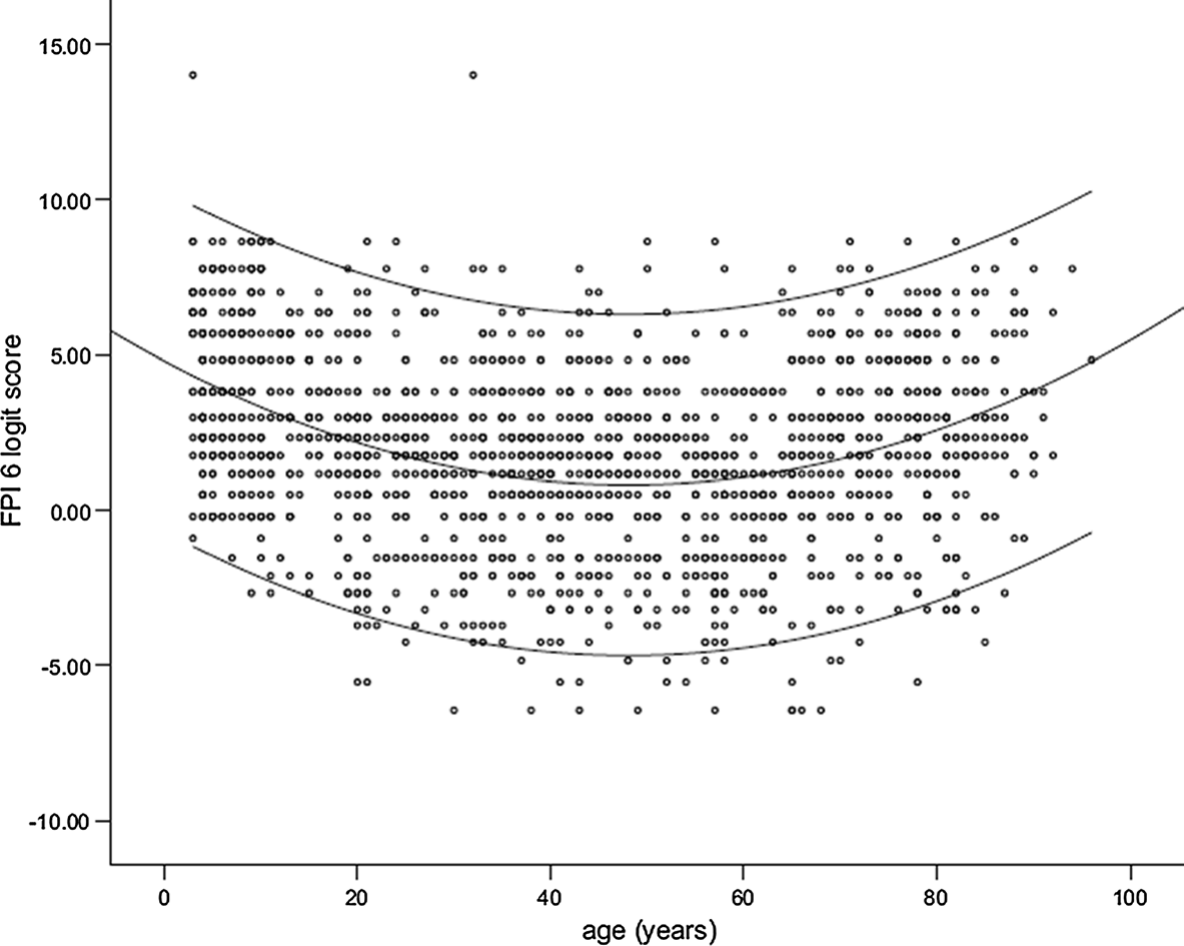
**The Scatter plot of FPI scores according to age, hence illustrate the normal presence of a flat foot posture in childhood, and reduction of the same with increasing age.** Reproduced from: Normative values for the Foot Posture Index, Redmond AC, Crane Y, Menz HB (2008), Journal of Foot and Ankle Research 1(6).

(i) *There should be a suitable test or examination.*Ideal screening tests have been described as having the following characteristics: simple, quick and easy to interpret; acceptable to the public (who participate voluntarily); accurate; repeatable, especially between observers; and sensitive and specific (rules the condition ‘in’ and ‘out’) [28]. It is easy to understand the appeal of foot-printing as a screening test for foot posture in children, as it is certainly quick, simple and at face value, easy to interpret. However the validity of foot printing to ascertain foot skeletal morphology is uncertain [29-31] if still frequently used [32-35]. Foot posture assessment in children has been less examined than in adult subjects, but the easiest and demonstrably reliable tools are the foot posture index (FPI-6) [31,36], from which ‘pronated’ cases (scores ≥ +6) can be identified and then classified and managed as directed by the paediatric flatfoot proforma (p-FFP) [22] (Figures 2,3 and 4). The range of statistically normal values needs to be appreciated by clinicians. Neither the FPI-6 nor the p-FFP has adequate sensitivity and specificity, which limits application.

### Treatment

(i) *There should be an accepted treatment for patients with recognised disease.*Is it either possible or necessary to prevent or reverse paediatric flatfoot? Given that normal physiological development sees reduced numbers of flatfoot posture with age, in a sense ‘reversible’–but as a part of its natural history. Given too, that it is from the commencement of the second decade of life, flatfoot posture is by definition less normal, this is perhaps the age group where prevention and reversibility are best focused.Harking back to the issues that may modulate the natural history of paediatric foot posture development, it is useful to look at these factors in terms of those that might be able to respond to external effect (viz treatment). The factors previously listed were: muscle tone, connective tissue quality, strength, genetic factors, antenatal growth, obesity, gender, inheritance, shoe use [27]. As a pragmatic approach, these factors have been categorised as ‘easy’ or ‘difficult’ to influence clinically. Strength [37,38], obesity/overweight [8,33], and shoe use [39] are the ‘easy’ factors which may be altered so as to possibly influence foot posture and resulting gait [8,33,37-39]. Muscle tone, connective tissue quality, genetic factors, antenatal growth, gender, and hereditary factors are ‘difficult’, if not impossible areas to change. Having said that, both hypotonia and connective tissue hypermobility may reduce with age [13]. However, the very issue of the need for treatment for paediatric flatfoot is tenuous, given the lack of supportive evidence for any asymptomatic cases, which form the majority [40]. Bluntly stated, if the paediatric flat foot cannot be definitively demonstrated to have definite and deleterious consequences in later life (when treatment can be instituted anyway), there may simply be no need for concern about the foot posture of healthy children. On the other hand, children who arrive at the end of their first life decade with very flatfeet, if usually asymptomatic, may be worth monitoring, strengthening, advising about footwear selection and overweight/obesity influence. Further research is required to determine which factors at which age, are associated with symptomatic flatfeet, such that any possible prevention may be reasonably employed [4,12]. Until this information is available the benefits of treatment of asymptomatic flatfeet remains questionable.

### Screening program

(i)  *There should be an agreed policy on whom to treat as patients.* It is agreed that children with painful flatfeet should be treated. It is also agreed that the ‘flat’ paediatric foot posture normally reduces with age across the first decade of life. It is further agreed that some conditions and groups with an underlying diagnosis have a greater propensity toward increased paediatric flatfoot deformity (e.g. cerebral palsy, Ehlers Danlos). The p-FFP designates treatment according to ‘traffic light’ colours: i.e. ‘red’ for painful flatfeet–treatment indicated; ‘yellow’ for children >8 to 10 years old with asymptomatic flatfeet–monitor, and perhaps use simple measures such as footwear support; ‘green’ for flatfeet in young children where this is the developmental ‘norm’ (not discounting young children diagnosable with wider and underlying diagnoses). There can be little contention about the ‘red’ and ‘green’ groups with respect to the respective need for treatment, and the normal physiological foot posture growth. However, the designated ‘yellow’ group has aroused considerable debate in some quarters [17,41-43], as has the findings of the Cochrane review which states that: “in the absence of symptoms there is no evidence to support the use of customised foot orthoses in asymptomatic children with flexible flatfeet” [12]. Whilst recent guidelines, assessment tools and a systematic review have consolidated much of the debate, there is as yet no truly universal policy on whom to treat, nor when or how to treat children with flexible flatfeet. Thorough assessment using the FPI-6 to filter, and the p-FFP to classify, would seem to be the best current approach.

(ii)  *Facilities for diagnosis and treatment should be available.* It is very difficult to justify any resources for wider diagnosis (let alone treatment) of paediatric flatfoot. The evidence is mounted against intervention of asymptomatic cases, which are the majority of clinical presentations. It is particularly difficult to justify screening programs for children less than 8 to 10 years of age, given the natural history of the resolution of ‘flat’ foot postures [26].

(iii)  *The cost of case-findings (including diagnosis and treatment of patients diagnosed) should be economically balanced in relation to possible expenditure on medical care as a whole.* Given the questionable relevance of the flexible paediatric flatfoot, and given the normal developmental profile of the same, it is unlikely that screening programs are ultimately cost-effective. The costs of failing to detect paediatric flatfoot are not evident, as most resolve physiologically over time, and those remaining will not definitely result in disability. The costs of screening programs will include staff time, equipment and the additional setting up procedures. There are also the subsequent wider health costs of referral for further evaluation and possible treatment (which may be nett cost versus gain, if unnecessary treatment occurs). Less measurable is the cost of parental concern that can be generated from receipt of evaluations designating their child as having “abnormal” feet and “requiring attention”. Most concerning of all is the unnecessary cost of any ‘automatic’ and subsequent treatment, especially customised foot orthoses for young, asymptomatic children–given the evidence against this approach.

(iv)  *Case-findings should be a continuing process and not a ‘once and for all’ project.* Isolated one-off screening programs at sites of convenience are unlikely to yield useful findings. This is what commonly happens with paediatric foot screenings, and as with any other screening programs, there is much less likelihood of valuable findings unless linked to a regular health surveillance project. Once again however, it is extremely difficult to see any justification for a specific foot component to paediatric health screenings, given that the most significant paediatric foot deformity, i.e. clubfoot, is detected at (or before) birth in developed countries [44,45].

## Comparison with developmental dysplasia of the hip (DDH)

The hips of newborn babies have long been routinely examined for dislocation, subluxation and instability. Developmental dysplasia of the hip (DDH) left uncorrected, is associated with long-term morbidity, including chronic pain, abnormal gait and arthritis [46]. Pertinent is that when detected early, DDH is very treatable with non-invasive, inexpensive methods (splinting, harnesses), with good results [47].

Applying the WHO principles of early disease detection to both paediatric DDH and flat feet provides clear comparison of both the need for, and benefits from, screening programs (Table 3). Screening for DDH adheres to the WHO principles, which justifies screening programs for the infant hip. This is not the case for asymptomatic flatfeet in children.

**Table 3:** The WHO principles of early disease detection applied to both developmental dysplasia of the hip (DDH) and paediatric flat foot posture

**WHO criteria**	**DDH**	**Flatfoot**
· The condition should be an important health problem.	Uncorrected DDH results in long-term pain, gait dysfunction and early arthritis	Indefinite prognosis for flexible flat feet, but rigid flat feet usually require treatment [48]
· There should be a recognisable latent or early symptomatic stage	Mostly detectable and reversible from birth	Most flat feet are asymptomatic in the first decade of life
· The natural history of the condition, including development from latent to declared disease should be adequately understood.	Some DDH normalizes in the first few months of life; controversy about when to treat, how long to monitor in cases of instability [49]. Recognisable risk factors in newborns are: breech birth, female, left hip affected, first born, family history of DDH [50].	A flat foot posture is expected in infants, and normally reduces with age. Recognisable associations with non-resolving flat feet may include: male, overweight or obesity, hypermobility, wider conditions e.g. Down’s, family history, increased shoe use from young age.
· There should be a suitable test or examination.	Clinical examination and ultrasound have demonstrated 97% sensitivity [51].	FPI-6 ≥ +6 indicates suitability of the p-FFP tool for diagnosis and directs management.
· The test should be acceptable to the population.	Clinical examination and ultrasound (and later x-rays) are acceptable tests.	No universally accepted definition for flat foot. [40].
· There should be an accepted treatment for patients with recognised disease.	Abduction splinting found to be safe and effective [52].	In the absence of symptoms, the indication for treatment is uncertain and should only be used when clinically definable outcomes can be improved.
· There should be an agreed policy on whom to treat as patients.	It is agreed that DDH be treated early to reduce the chance of serious pathology. There is some controversy regarding the age to commence treatment, given that some cases resolve.	The best available evidence supports treating rigid or symptomatic flexible flatfeet [48]. There is no clear evidence to support the treatment of most asymptomatic cases, especially in younger children [25]
· Facilities for diagnosis and treatment should be available.	Clinical examination and ultrasound are readily available.	Observation, the FPI-6 and the p-FFP are readily and freely available measures.
· The cost of case-findings (including diagnosis and treatment of patients diagnosed) should be economically balanced in relation to possible expenditure on medical care as a whole.	Late diagnosis increases worse outcome and increased need for surgery [46]	Not supported
· Case-findings should be a continuing process and not a ‘once and for all’ project.	Routinely occurs from birth and early paediatric health checks.	Not indicated

## Conclusions

Despite the regular occurrence of podiatry screenings for children (with focus of foot posture), there is no good evidence to deem such activities as warranted, given both the natural history of paediatric foot morphology, the largely asymptomatic presentation, and the lack of evidence to support treatment. The application of the WHO criteria to justify screenings, clearly negate the need for such practice, and until there is evidence to the contrary, podiatry screenings for children not related to formalized research protocols, appear neither necessary nor appropriate.

## Competing interests

The author declares no competing interests, either financial or non-financial.

## References

[B1] Jordan KP Kadam UT Hayward R Porcheret M Young C Croft P Annual consultation prevalence of regional musculoskeletal problems in primary care: an observational study BMC Musculoskelet Disord 2010 11 144 10.1186/1471-2474-11-144 20598124 PMC2903510

[B2] Staheli LT Evaluation of planovalgus foot deformities with special reference to the natural history J Am Podiatr Med Assoc 1987 77 2 6 3820110 10.7547/87507315-77-1-2

[B3] El O Akcali O Kosay C Kaner B Arslan Y Sagol E Soylev S Iyidogan D Cinar N Peker O Flexible flatfoot and related factors in primary school children: a report of a screening study Rheumatol Int 2006 26 1050 1053 10.1007/s00296-006-0128-1 16670858

[B4] Evans AM Rome K A Cochrane review of the evidence for non-surgical interventions for flexible pediatric flat feet Eur J Phys Rehabil Med 2011 47 69 89 21448121

[B5] Wilson JMG Jungner G Principles and practice of screening for disease. 34 1968 World Health Organisation, Geneva

[B6] Wright MC Colville DJ Oberklaid F Is community screening for amblyopia possible, or appropriate? Arch Dis Child 1995 73 192 195 10.1136/adc.73.3.192 7492153 PMC1511287

[B7] Shapiro J Rhee CS Podiatry screening project for children in the District of Columbia Public Health Reports 1970 85 803 808 10.2307/4593969 4989474 PMC2031768

[B8] Pfeiffer M Kotz R Ledl T Hauser G Sluga M Prevalence of flat foot in preschool-aged children Pediatrics 2006 118 634 639 10.1542/peds.2005-2126 16882817

[B9] Australian Health Ministerial Advisory Council Population Based Screening Framework 2008 http://www.cancerscreening.gov.au/internet/screening/publishing.nsf/Content/pop-based-screening-fwork/

[B10] Handbook–tuberculosis 2012 http://www.health.gov.au/internet/immunise/publishing.nsf/Content/Handbook-tuberculosis

[B11] Cancer council Screening position statement 2012 http://www.cancer.org.au/policy/positionstatements.htm

[B12] Rome K Ashford RL Evans AM Non-surgical interventions for paediatric pes planus Cochrane Database of Systematic Reviews 2007 1 7 Art. No.: CD006311 10.1002/14651858.CD006311 20614443

[B13] Murray KJ Hypermobility disorders in children and adolescents Best Pract Res Clin Rheumatol 2006 20 329 351s 10.1016/j.berh.2005.12.003 16546060

[B14] Mauch M Grau S Krauss I Maiwald C Horstmann T Foot morphology of normal, underweight and overweight children Int J Obes (Lond) 2008 32 1068 1075 10.1038/ijo.2008.52 18414422

[B15] Simmonds JV Keer RJ Hypermobility and the hypermobility syndrome Man Ther 2007 12 298 309s 10.1016/j.math.2007.05.001 17643337

[B16] Selby-Silverstein L Hillstrom HJ Palisano RJ The effect of foot orthoses on standing foot posture and gait of young children with Down Syndrome NeuroRehabilitation 2001 16 193 11790903

[B17] Evans AM The flat-footed child–to treat or not to treat, what is the clinician to do? J Am Podiatr Med Assoc 2008 98 386 393 18820042 10.7547/0980386

[B18] Yeagerman SE Cross MB Positano R Doyle SM Evaluation and treatment of symptomatic pes planus Curr Opin Pediatr 2011 23 60 67 10.1097/MOP.0b013e32834230b2 21169838

[B19] Blitz NM Stabile RJ Giorgini RJ DiDomenico LA Flexible pediatric and adolescent pes planovalgus: conservative and surgical treatment options Clin Podiatr Med Surg 2010 27 59 77 10.1016/j.cpm.2009.09.001 19963170

[B20] Harris EJ The natural history and pathophysiology of flexible flatfoot Clin Podiatr Med Surg 2010 27 1 23 10.1016/j.cpm.2009.09.002 19963167

[B21] Garcia-Rodriguez A Martin-Jimenez F Carnero-Varo M Gomez-Gracia E Gomez-Aracena J Fernandez-Crehuet J Flexible flat feet in children: a real problem? Pediatrics 1999 103 e84 10.1542/peds.103.6.e84 10353981

[B22] Evans AM Nicholson H Zakaris N The paediatric flat foot proforma (p-FFP): improved and abridged following a reproducibility study J Foot Ankle Res 2009 2 25 10.1186/1757-1146-2-25 19691841 PMC2734539

[B23] Shepherd RB Physiotherapy in paediatrics 1995 3 Butterworth Heinemann, Oxford

[B24] Gould N Moreland M Alvarez R Trevino S Fenwick J Development of the child’s arch Foot Ankle 1989 9 241 245 2731836 10.1177/107110078900900506

[B25] Gilmour JC Burns Y The measurement of the medial longitudinal arch in children Foot Ankle Int 2001 22 493 498 11475457 10.1177/107110070102200607

[B26] Staheli LT Planovalgus foot deformity. Current status J Am Podiatr Med Assoc 1999 89 94 99 10063780 10.7547/87507315-89-2-94

[B27] Evans AM Paediatrics. The pocket podiatry guide 2010 1 Churchill Livingstone Elsevier

[B28] Cochrane A Holland W Validation of screening procedures Br Med Bull 1971 27 3 8 5100948 10.1093/oxfordjournals.bmb.a070810

[B29] Kanatli U Yetkin H Cila E Footprint and radiogeaphic analysis of the feet J Pediatr Orthop 2001 21 225 228 11242255

[B30] Evans AM The paediatric flat foot and general anthropometry in 140 Australian school children aged 7–10 years J Foot Ankle Res 2011 4 12 10.1186/1757-1146-4-12 21513507 PMC3102032

[B31] Redmond AC Crane YZ Menz HB Normative values for the Foot Posture Index J Foot Ankle Res 2008 1 6 10.1186/1757-1146-1-6 18822155 PMC2553778

[B32] Villarroya MA Esquivel JM Tomás C Moreno LA Buenafé A Bueno G Assessment of the medial longitudinal arch in children and adolescents with obesity: footprints and radiographic study Eur J Pediatr 2009 168 559 567 10.1007/s00431-008-0789-8 18751725

[B33] Mickle KJ Steele JR Munro BJ The feet of overweight and obese young children: are they flat or fat? Obesity 2006 14 1949 1953 10.1038/oby.2006.227 17135610

[B34] Lin C Lee H Chen J Lee H Kuo M Development of a quantatative assessment system for correlation analysis of footprint parameters to postural control in children Physiol Meas 2006 27 119 130s 10.1088/0967-3334/27/2/003 16400199

[B35] Chen JP Chung MJ Wang MJ Flatfoot prevalence and foot dimensions of 5- to 13-year-old children in taiwan Foot Ankle Int 2009 30 326 332 10.3113/FAI.2009.0326 19356357

[B36] Redmond AC Crosbie J Ouvrier R Development and validation of a novel rating system for scoring foot posture: the Foot Posture Index Clin Biomech 2006 21 89 98 10.1016/j.clinbiomech.2005.08.002 16182419

[B37] Rose KJ Burns J North KN Relationship between foot strength and motor function in preschool-age children Neuromuscul Disord 2009 19 104 107 10.1016/j.nmd.2008.10.007 19056267

[B38] Riccio I Gimigliano F Porpora G Iolascon G Rehabilitative treatment in flexible flatfoot: a perspective cohort study Musculoskelet Surg 2009 93 101 107 10.1007/s12306-009-0037-z 19777377

[B39] Wegener C Hunt AE Vanwanseele B Burns J Smith RM Effect of children;s shoes on gait: a systematic review and meta-analysis J Foot Ankle Res 2011 4 3 10.1186/1757-1146-4-3 21244647 PMC3031211

[B40] Rome K Ashford RL Evans AM Non-surgical interventions for paediatric pes planus (review) Cochrane Database Syst Rev 2010 7 10.1002/14651858.CD006311.pub2 20614443

[B41] D'Amico JC The flat-footed child–to treat or not to treat: what is the clinician to do? letter to the editor J Am Podiatr Med Assoc 2009 99 267 19448182 10.7547/0980267

[B42] Bresnahan P The flat-footed child–to treat or not to treat. What is the clinician to do? J Am Podiatr Med Assoc 2009 99 178 19299359 10.7547/0980178

[B43] Evans AM The flat-footed child–to treat or not to treat. What is the clinician to do? J Am Podiatr Med Assoc 2009 99 179 10.7547/0980267 19448182

[B44] McElroy T Konde-Lule J Neema S Gitta S Understanding the barriers to clubfoot treatment adherence in Uganda: a rapid ethnographic study Disabil Rehabil 2007 29 845 855 10.1080/09638280701240102 17577719

[B45] Ponseti IV Morecuende JA Mosca V Pirani S Dietz FR Herzenberg JE Clubfoot: ponseti management. second 2003 Global-HELP Publication, Staheli, L. T

[B46] Shorter D Hong T Osborn DA Screening programmes for developmental dysplasia of the hip in newborn infants Cochrane Database Syst Rev 2011 7 CD004595 21901691 10.1002/14651858.CD004595.pub2 PMC6464894

[B47] Cady RB Developmental dysplasia of the hip: definition, recognition, and prevention of late sequelae Pediatr Ann 2006 35 92 101 16493916 10.3928/0090-4481-20060201-09

[B48] Harris EJ Vanore JV Thomas JL Kravitz SR Mendicino RW Silvani SH Gassen SC Diagnosis and treatment of pediatric flatfoot J Foot Ankle Surg 2004 43 341 373 10.1053/j.jfas.2004.09.013 15605048

[B49] Woolacott NF Puhan MA Steurer J Kleijnen J Ultrasonography in screening for developmental dysplasia of the hip in newborns: systematic review Br Med J 2005 10.1136/bmj.38450.646088.E0 PMC558371 15930025

[B50] Ortiz-Neira CL Paolucci EO Donnon T A meta-analysis of common risk factors associated with the diagnosis of developmental dysplasia of the hip in newborns Eur J Radiol 2012 81 344 351 10.1016/j.ejrad.2011.11.003 22119556

[B51] Dogruel H Atalar H Yavuz OY Sayli U Clinical examination versus ultrasonography in detecting developmental dysplasia of the hip Int Orthop 2008 32 415 419 10.1007/s00264-007-0333-x 17333184 PMC2323411

[B52] Kitoh H Kawasumi M Ishiguro N Predictive factors for unsuccessful treatment of developmental dysplasia of the hip by the Pavlik harness J Pediatr Orthop 2009 29 552 557 10.1097/BPO.0b013e3181b2f200 19700982

